# Crop Functional Genomics and Biological Breeding—2nd Edition

**DOI:** 10.3390/plants15081195

**Published:** 2026-04-13

**Authors:** Guangan Hu, Jie Huang, Jiezheng Ying, Jian Zhang, Yifeng Wang

**Affiliations:** State Key Laboratory of Rice Biology and Breeding, China National Rice Research Institute, Hangzhou 311400, China; 13332564410@163.com (G.H.); huangjie@caas.cn (J.H.); yingjiezheng@caas.cn (J.Y.); zhangjian@caas.cn (J.Z.)

## 1. Introduction

Functional genomics serves as a critical link between genotype and phenotype, with its core mission being to elucidate gene functions and their regulatory networks at the genome-wide level. Unlike traditional reverse genetics, functional genomics leverages high-throughput sequencing, bioinformatics, and multi-omics integration to systematically reveal the genetic basis of complex agronomic traits, enabling breeders to comprehensively understand the mechanisms shaping crop yield, resistance, quality, and other traits from a genomic perspective [1]. However, technological means are ultimately tools; the fundamental goal of functional genomics is to serve breeding practices—by mining key genes and dissecting regulatory networks to provide precise operational targets for biological breeding, thereby efficiently translating foundational research outcomes into applicable breeding resources [2].

Currently, global agriculture faces multiple challenges, including continuous population growth, frequent extreme weather events, and increasing abiotic and biotic stresses, such as soil salinization, drought, pests, and diseases, alongside diminishing arable land resources. Against this backdrop, developing new crop varieties with high, stable yields and superior quality has become a crucial pathway to ensuring food security and promoting sustainable agricultural development. As a cutting-edge technology in modern agriculture, biological breeding is rapidly evolving from traditional empirical breeding to precision, molecular-design breeding. Functional genomics serves as the core driving force for biological breeding. It not only reveals the genes and their regulatory networks controlling key traits but also provides direct operational targets for modern breeding technologies such as molecular marker-assisted selection, gene-editing-based breeding, and genomic selection [3].

Building on this foundation, the Special Issue “Crop Functional Genomics and Biological Breeding—2nd Edition” in *Plants* continues to focus on recent advancements in this field. It compiles 11 research articles covering major staple crops such as rice, maize, wheat, and soybean, as well as important cash crops such as rapeseed, Zanthoxylum, and bitter gourd. Across these 11 studies, distinct commonalities emerge: research paradigms, the widespread application of multi-omics integration strategies, the close integration of gene function elucidation with breeding value exploration, and coverage spanning abiotic, biotic, and combined stresses. These fully reflect the current trend in functional genomics research towards systematization and application-oriented research. Collectively, these studies demonstrate a complete chain from gene mapping and cloning to breeding application, closely revolving around the three pillars of modern crop improvement: yield, resistance, and quality.

## 2. Yield-Related Gene Mining and Breeding Utilization

Regarding the genetic dissection of yield-related traits, two studies included in this Special Issue exemplify how functional genomics provides the core impetus for biological breeding. These two studies illustrate pathways for deep integration of genomic technologies with breeding practices, focusing on the regulation of agronomic traits and the evolution of polyploid genomes, respectively.

The leaf emergence rate is a key factor determining plant architecture, biomass accumulation, and final seed yield in rapeseed. However, its molecular regulatory mechanism has long remained unclear, limiting the molecular breeding of related traits. Qin et al. systematically dissected the rapid leaf emergence trait in *Brassica napus* using a functional genomics strategy that combined genetic analysis, BSA-seq, and transcriptome sequencing [4]. The study revealed that this trait is controlled by two additive–dominant major genes (2MG-EAD), a conclusion validated by genetic analysis over two consecutive years, indicating a relatively stable genetic basis. Further, BSA-seq identified three novel genomic intervals on chromosomes A02 and A04. Integrating transcriptome data enabled the refined screening of genes within these intervals, ultimately pinpointing 10 candidate genes. Notably, these candidates include not only known key regulators of development, such as *FT* and *RGL1*, but also several CLE-related genes (*CLE4*, *CLE6*, and *CLE7*), suggesting a significant role for the CLE signalling pathway in regulating leaf emergence rate. From a biological breeding perspective, this study not only provides new insights into the regulatory network of leaf development in rapeseed but, more importantly, offers actionable breeding resources-the identified candidate genes can be directly applied in molecular marker-assisted selection for developing high-yield rapeseed varieties with optimized plant architecture.

From a broader genomic perspective, a crucial mission of functional genomics is to unravel the evolutionary patterns of complex genomes, thereby laying the theoretical foundation for the use of polyploid crops in breeding. Wheat, a typical allohexaploid, exhibits significant functional differentiation among its A, B, and D over long-term evolution, an asymmetry with profound implications for unlocking breeding potential. Zhu et al. systematically identified, classified, and analyzed the evolutionary dynamics of pseudogenes in the wheat genome at the whole-genome level using a reference genome [5]. Once considered non-functional “junk” DNA, functional genomics research indicates that pseudogenes may harbour important functions in genome evolution and regulatory network remodelling, representing potential sources of heritable variation for breeding. This study revealed significant asymmetry among the three subgenomes in terms of pseudogene count, distribution, evolutionary dynamics, and selective constraints: the B subgenome contained significantly more pseudogenes, and its evolutionary patterns differed markedly from those of the A and D subgenomes. Further analysis found that pseudogene formation is closely linked to transposable elements. Still, the timing of their formation did not perfectly align with ancient genomic upheavals in the wheat lineage, suggesting the presence of more complex regulatory mechanisms. Functional enrichment analysis showed that the parental genes of pseudogenes are primarily enriched in non-core functions and tissue-specific expression. From a breeding standpoint, this research offers a new perspective on gene redundancy and divergence in polyploid genomes, providing a theoretical basis for exploiting “hidden” genetic variation in polyploid crops and long-term significance for expanding the pool of usable genetic resources for wheat breeding.

Synthesizing these two studies, the dual value of functional genomics in yield breeding becomes clear. On one hand, through modern genomic technologies like BSA-seq and transcriptomics, key agronomic traits are finely mapped and candidate genes screened, directly providing operational targets for molecular breeding—the study on rapeseed leaf emergence rate exemplifies this approach, with identified candidates like *FT*, *RGL1*, and *CLE* offering resources for plant architecture improvement. On the other hand, by systematically analyzing the evolutionary history of polyploid crop genomes, potentially useful variations within complex genomes are uncovered, providing theoretical support for long-term breeding strategies—the wheat pseudogene study exemplifies this approach. Methodologically, both studies reflect significant trends in current functional genomics: the former showcases the efficiency of combining BSA-seq with transcriptome analysis for mining genes controlling agronomic traits. At the same time, the latter demonstrates a paradigm that combines large-scale genomic data with evolutionary biology analysis. Ultimately, both serve the core goal of providing actionable resources and strategies for biological breeding.

Looking ahead, the application of functional genomics in yield breeding should further deepen, including enhancing allelic variation analysis of candidate genes within breeding populations, using gene editing to create superior alleles, and deeply exploring the breeding potential arising from subgenomic asymmetry in polyploid crops. It is anticipated that, with continued advances in functional genomics research, the molecular regulatory networks governing yield-related traits will become increasingly clear, leading to more precise and efficient applications in biological breeding and thereby providing robust scientific support for achieving high-yield crop breeding goals.

## 3. Resistance Gene Dissection and Breeding Application

In the realm of resistance breeding, several studies in this Special Issue demonstrate the value of functional genomics in dissecting crop stress response mechanisms from multiple angles, including salt, cold, and combined stresses, as well as disease resistance.

Salt stress is a key factor limiting crop production on saline–alkali land. Shi et al. identified the NAC transcription factor *GmNAC035* in soybean, which is rapidly induced by salt and abscisic acid [6]. Overexpressing *GmNAC035* in *Arabidopsis* enhanced salt tolerance. The mechanism involved activating the SOS pathway, calcium signalling, and ABA-responsive genes while reducing oxidative damage. The study also found significant upregulation of stress-responsive genes, such as *AtRD29B* and *AtDREB2A*, in overexpressing plants, indicating that GmNAC035 enhances salt tolerance by coordinating multiple pathways. Similarly, Wang et al. identified the R2R3-MYB transcription factor *BnaMYB73* in rapeseed, whose expression was strongly induced by salt and osmotic stress [7]. Transgenic *Arabidopsis* overexpressing *BnaMYB73* exhibited higher proline accumulation, increased superoxide dismutase (SOD) and peroxidase (POD) activities under stress, along with upregulation of stress-responsive genes like *AtRD29B* and *AtDREB2A*, suggesting a conserved salt tolerance function for this transcription factor across species. Fan et al. provided a comprehensive review systematically outlining saline–alkali tolerance mechanisms from model plants to staple crops [8]. They compared the conservation and species-specificity of the SOS signalling pathway, osmotic regulation, and reactive oxygen species (ROS) scavenging in *Arabidopsis*, rice, maize, and wheat, proposing a “crop-centric” breeding strategy that offers important insights for future saline–alkali tolerant breeding.

Low-temperature stress is another major limiting factor for crop production. Zhou et al. investigated the chilling tolerance mechanism during maize germination, finding that tolerant inbred lines more strongly activated the phenylpropanoid and flavonoid biosynthesis pathways under low temperature [9]. Physiological analysis showed that tolerant lines accumulated less malondialdehyde (MDA) and exhibited higher SOD and POD activities under chilling stress. Among the identified genes, *ZmPER5*, encoding a class III peroxidase, was significantly upregulated in tolerant lines, potentially contributing to chilling tolerance by enhancing cell wall structure and ROS scavenging capacity. Other lignin- and flavonoid-related genes also showed genotype-specific induction patterns consistent with chilling tolerance. In bitter gourd, a thermophilic vegetable crop sensitive to low temperature, Yang et al. identified a low-temperature-responsive NAC transcription factor, *McNAC087* [10]. Transcriptome analysis revealed significant expression differences in *McNAC087* under cold stress between tolerant and sensitive inbred lines. Subcellular localization confirmed that McNAC087 localizes to the nucleus. Overexpressing *McNAC087* in *Arabidopsis* enhanced cold tolerance by activating the ICE-CBF-COR cascade pathway and boosting antioxidant capacity, manifested by increased proline accumulation and higher antioxidant enzyme activities. It reduced MDA and ROS levels, providing a candidate gene for improving bitter gourd’s cold tolerance.

In terms of disease resistance, two studies demonstrated the potential of functional genomics to identify resistance genes. Fan et al. performed a systematic analysis of the Dof transcription factor family in soybean, identifying 78 *GmDof* genes distributed across 19 chromosomes [11]. Phylogenetic analysis categorized these genes into nine subfamilies. Subcellular localization analysis indicated that most GmDof proteins are nuclear-localized. Through RNA-seq and qRT-PCR screening, *GmDof63* was found to be specifically induced upon infection by *Phytophthora sojae* and strongly induced by salicylic acid (SA) and ethylene (ETH) treatments. Soybean plants overexpressing *GmDof63* showed enhanced resistance to Phytophthora root rot, accompanied by significant upregulation of several pathogenesis-related (PR) genes. Furthermore, the promoters of these PR genes contained numerous AAAG or TTTC motifs, suggesting that GmDof63 may directly or indirectly regulate their expression, thereby participating in the soybean defence response against *P. sojae*. In rice, Sanglou et al. characterized the spotted leaf mutant HM113, which exhibits spontaneous lesion formation and enhanced resistance to bacterial blight [12]. Through map-based cloning and CRISPR/Cas9 gene editing, the study confirmed that a single nucleotide substitution (A847T) in a gene encoding a cysteine-rich receptor-like kinase (CRK), resulting in a serine-to-cysteine change, was responsible for the phenotype. Introducing a frameshift mutation upstream of the A847T site using CRISPR/Cas9 eliminated the spotted leaf phenotype and abolished bacterial blight resistance, indicating that this gene acts as a gain-of-function resistance gene. This study provides a new exploitable allele for breeding rice disease resistance.

Under field conditions, crops often face multiple stresses simultaneously. Cao et al. explored the synergistic responses of rice seedlings to brown planthopper (BPH) infestation and high-temperature stress [13]. Interestingly, high-temperature pretreatment enhanced rice resistance to BPH, while BPH infestation did not affect rice tolerance to high temperature. Transcriptome analysis indicated that differentially expressed genes induced by high temperature were enriched in metabolic processes and the phenylpropanoid biosynthesis pathway, potentially linked to the formation of cross-tolerance. Using weighted gene co-expression network analysis (WGCNA), the study further identified two modules, magenta and black, associated with biological processes such as protein folding and transmembrane transport, and screened candidate genes potentially regulating responses to both stresses. This research emphasizes the importance of investigating combined stresses and provides new genetic resources for developing multi-resistant rice varieties.

Looking across these resistance studies, the core value of functional genomics in stress-resistance breeding is clearly evident. From single stress to combined stress, and from staple to specialty crops, these studies demonstrate the powerful efficacy of multi-omics integration strategies in dissecting crop. mechanisms. Through genome-wide identification, phylogenetic analysis, expression profiling, and transgenic functional validation, researchers have not only revealed the molecular regulatory networks underlying responses to salt stress, cold stress, pathogen infection, and combined high-temperature insect stress in crops, but also identified key gene resources with potential for breeding applications. Particularly noteworthy is the study on combined stress, reflecting the deepening of functional genomics research from single stressors towards multifactorial interactions, which more closely aligns with actual field production demands. These research outcomes provide important targets for molecular marker-assisted selection and gene-editing-based breeding and lay a solid foundation for developing new crop varieties with multiple resistance and broad adaptability.

## 4. Genetic Regulation and Improvement of Quality Traits

Quality improvement constitutes one of the three pillars of modern breeding, directly impacting the commercial value and market competitiveness of agricultural products. One study featured in this Special Issue focuses on the speciality crop Zanthoxylum and employs a multi-omics joint analysis strategy to uncover key genes involved in coumarin biosynthesis, providing new insights for the genetic improvement of quality traits.

Coumarin compounds are crucial sources of the characteristic numbing and aromatic flavours in Zanthoxylum pericarp; their content and composition directly affect flavour quality and commercial value. Chen et al. studied two Zanthoxylum varieties, Z. bungeanum (red prickly ash) and Z. armatum (green prickly ash, known as Tengjiao), systematically analyzing mature pericarps using widely targeted metabolomics and transcriptome sequencing [14]. The study detected 583 metabolites, with flavonoids, phenolic acids, and alkaloids as the main components. Twenty-four coumarin compounds were identified, with most showing significantly higher content in Z. armatum compared to Z. bungeanum. By jointly analyzing metabolomic and transcriptomic data, the study further identified 56 candidate genes highly correlated with the accumulation of scopoletin and scopolin. Functional validation experiments demonstrated that transient overexpression of *CCoAOMT*, *COMT*, and *F6H* genes in *Nicotiana benthamiana* significantly increased scopoletin and scopolin content, confirming the crucial roles of these genes in coumarin biosynthesis.

This study fully demonstrates the efficiency of integrating metabolomic and transcriptomic strategies in quality trait research. Compared to traditional single-omics analysis, multi-omics joint analysis captures both metabolite accumulation and gene expression changes simultaneously, allowing rapid identification of key regulatory genes through correlation analysis and significantly shortening the timeline from trait dissection to gene identification. This research paradigm applies not only to Zanthoxylum but also provides a methodological framework for quality improvement in other speciality crops, such as tea plants, medicinal herbs, and speciality fruits and vegetables.

From a broader perspective, research on the genetic regulation of quality traits is progressively moving from traditional analyses of single traits to a new stage characterized by multi-omics integration and the systematic dissection of metabolic pathways. In staple crops like rice, maize, and wheat, regulatory networks for quality-related traits such as starch synthesis, protein accumulation, and lipid metabolism are being progressively elucidated, providing important targets for molecular design breeding [15,16]. However, for speciality crops such as Zanthoxylum, tea, and medicinal herbs, genetic research on quality traits lags, with significant gaps in understanding the mechanisms of synthesis for flavour compounds and active ingredients. The study on Zanthoxylum included in this Special Issue represents an active exploration in this context, and its multi-omics integration paradigm can serve as a methodological reference for quality improvement in other speciality crops.

It is worth noting that quality improvement in speciality crops is of significant practical importance. With consumption upgrading and diversifying market demands, consumer focus on agricultural products has shifted from solely yield to comprehensive qualities, including flavour, nutrition, and functionality. The research on coumarins in Zanthoxylum not only helps elucidate the synthesis mechanism of the numbing flavour compounds but also provides gene resources for targeted improvement of Zanthoxylum flavour quality, underscoring the unique value of functional genomics research in meeting diversified market demands. In the future, efforts should be intensified to advance the genetic dissection of quality traits in speciality crops, and to promote the integrated application of metabolic engineering and gene editing technologies for quality improvement, thereby providing scientific support to meet diverse market needs.

## 5. Conclusions and Perspectives

The Special Issue “Crop Functional Genomics and Biological Breeding—2nd Edition” compiles 11 cutting-edge research articles that systematically showcase the central role of functional genomics in crop improvement. These studies closely align with the three pillars of modern crop breeding—yield, resistance, and quality—and cover staple crops such as rice, maize, wheat, and soybean, as well as speciality crops such as rapeseed, Zanthoxylum, and bitter gourd. Collectively, they illustrate a complete chain from gene discovery to breeding application.

Looking across these 11 studies, several important trends in current crop functional genomics research are evident. Firstly, multi-omics integration has become a powerful paradigm for dissecting complex traits—the combined application of genomics, transcriptomics, metabolomics, BSA-seq, WGCNA, and other techniques greatly enhances the efficiency and precision of gene discovery. Secondly, research perspectives are expanding from single stresses to combined stresses; the study on the synergistic response of rice to high temperature and brown planthopper provides a model for addressing multifactorial interactions in the field. Thirdly, the distance from gene discovery to breeding application is shortening; several candidate genes featured in this Special Issue already have potential for use in molecular marker-assisted selection or gene-editing-based breeding, reflecting the tangible driving force of functional genomics for biological breeding.

Looking ahead, research in crop functional genomics should continue to focus on several directions. First, enhance allelic variation analysis of candidate genes within breeding populations and use gene-editing technologies to create superior alleles, accelerating the translation from gene discovery to variety improvement. Second, deepen research on responses to combined stresses. In the context of climate change, multiple stresses, such as high temperatures, drought, salinity, pests, and diseases, often occur simultaneously or sequentially. Dissecting the mechanisms underlying crop synergistic responses to multifactorial interactions is crucial for developing widely adaptable varieties. Third, expand functional genomics research on speciality crops. Explorations of crops such as Zanthoxylum and bitter gourd indicate that speciality crops harbour abundant genetic resources for quality and resistance, warranting further exploration to meet diversified market demands. Fourth, promote the deep integration of multi-omics data with intelligent breeding technologies. Leveraging artificial intelligence and machine learning algorithms to construct genotype-phenotype prediction models can facilitate the leap from gene discovery to variety selection.

We believe these research outcomes not only deepen our understanding of the mechanisms underlying crop yield, resistance, and quality formation but also provide directly applicable gene resources and technical solutions for biological breeding. The deep integration of functional genomics and biological breeding will provide robust scientific support for addressing global climate change, ensuring food security, and promoting the sustainable development of green agriculture.

## Data Availability

No new data were created or analyzed in this study. Data sharing is not applicable to this article.
